# COVID-19 and diabetes: the contributions of hyperglycemia

**DOI:** 10.1093/jmcb/mjaa054

**Published:** 2020-10-01

**Authors:** Jing Wang, Wen Meng

**Affiliations:** National Clinical Research Center for Metabolic Diseases, Metabolic Syndrome Research Center, and Department of Metabolism and Endocrinology, The Second Xiangya Hospital of Central South University, Changsha 410011, Hunan, China

Coronavirus disease 2019 (COVID-19) caused by coronavirus SARS-CoV-2 infection has now evolved into a worldwide crisis that triggers substantial morbidity and mortality. COVID-19 occurs more frequently and has more serious complications in patients with diabetes mellitus, but the underlying mechanisms remain largely elusive. Here, we summarize current and evolving concepts on the detrimental effect of hyperglycemia on SARS-CoV-2 infection and consequences, focusing on several key mechanisms underlying the link between diabetes and COVID-19. A better understanding of the mechanisms by which hyperglycemia worsens the prognosis of COVID-19 is critical for reducing the risk of SARS-CoV-2 infection and its associated mortality.

## Introduction

COVID-19 caused by coronavirus SARS-CoV-2 infection was declared by the World Health Organization on March 11, 2020 as a global pandemic. Clinical data show that the severe cases can rapidly progress to acute respiratory distress syndrome (ARDS), septic shock, and multiple organ dysfunction syndrome (MODS) (Guan et al., 2020). Importantly, recent studies find that advanced age or underlying medical comorbidities, such as diabetes mellitus, hypertension, cardiovascular diseases, and acute kidney injury, have a demonstrated higher risk for developing more severe cases, as well as suffering a higher risk of mortality (Li et al., 2020b).

Epidemiologic evidences show that type 2 diabetes mellitus (T2DM) is the second most common comorbidity of COVID-19, and that people with T2DM are more susceptible to SARS-CoV-2 infection (Li et al., 2020b; Muniyappa and Gubbi, 2020). It is reported that ARDS is the major cause for mortality of COVID-19 patients, which is more prevalent in COVID-19 patients with pre-existing diabetes (Guan et al., 2020). A great amount of data worldwide reveal that COVID-19 patients with hyperglycemia or T2DM have a greatly enhanced release of inflammatory cytokines or the cytokine storm syndrome, which leads to immunosuppression and multi-organ failure (Ye et al., 2020), providing further evidence on a potential link between T2DM and COVID-19 due to inflammation and immune deficiency. As the global pandemic is still growing, if we resolve the questions of key pathways and mechanisms underlying the link between T2DM and COVID-19, it would be tremendously helpful for treating severe patients with COVID-19 worldwide.

## Hyperglycemia: a key relationship of COVID-19 and diabetes

A two-center retrospective study was performed at two tertiary hospitals in Wuhan, China including 1561 patients with COVID-19, representing that a higher proportion of intensive care unit (ICU) admission (17.6% vs. 7.8%, *P *=* *0.01) and more fatal cases (20.3% vs. 10.5%, *P *=* *0.017) were identified in COVID-19 patients with diabetes (Apicella et al., 2020). In addition, the prevalence of diabetes in 27955 Italian patients who died from COVID-19 is 31.1% (Apicella et al., 2020). In the UK, a survey of 23804 patients died from COVID-19 shows the prevalence of T2DM of 32% and T1DM of 1.5%, respectively (Apicella et al., 2020). In summary, COVID-19 patients with pre-existing diabetes have a worse prognosis, and the mechanisms may be complicated.

Diabetes mellitus, especially T2DM, is a metabolic disease characterized by abnormally hyperglycemia due to impaired insulin secretion and/or insulin action (Dalmas, 2019). Accumulating evidence in the COVID-19 pandemic shows that hyperglycemia could increase the risk of mortality in patients with COVID-19 (Bode et al., 2020). Indeed, elevated fasting blood glucose (≥7.0 mmol/L) or acute uncontrolled hyperglycemia (defined as blood glucose >10 mmol/L twice within any 24 h period) is related to morbidity and/or mortality from COVID-19 (Yang et al., 2020). In addition, a recent study shows that among ∼7300 cases of COVID-19, T2DM is associated with a higher death rate (Zhu et al., 2020). However, the death rate is greatly reduced in diabetic patients with better controlled blood glucose levels (Zhu et al., 2020). These findings suggest that hyperglycemia in the early phase of COVID-19 may play an important role in determining the seriousness of the prognosis. It is also reported that the prevalence of diabetes in 1590 Chinese patients with COVID-19 rose to 34.6%, while for general people it was 8.2% in China (Apicella et al., 2020). Together, people with diabetes are more susceptible to SARS-CoV-2 infection, and SARS-CoV-2 infection may increase the level of blood glucose, suggesting that hyperglycemia is a key factor between COVID-19 and diabetes. Recently, it is reported that insulin infusion may be an effective method for achieving glycemic targets and improving clinical outcome of COVID-19 (Sardu et al., 2020). Therefore, well-controlled glycemia is important for improving outcomes of patients with COVID-19 and pre-existing T2DM.

## Potential mechanisms of hyperglycemia-caused worse prognosis for COVID-19

A number of possible mechanisms have been proposed to date to explain the phenomenon that people with diabetes or hyperglycemia appear to have increased risk of SARS-CoV-2 infection rate and worse consequences after infection. Understanding the mechanisms is critical for diabetes patients and healthcare professionals to manage and reduce the risk of SARS-CoV-2 infection during the COVID-19 pandemic.

### Increased ACE2 receptor in epithelial cell membrane and ACE2 receptor glycosylation

It is now well known that SARS-CoV-2 infects host cells through the angiotensin-converting enzyme 2 (ACE2) receptor, a plasma membrane protein expressed largely in lungs, leading to COVID-19-related interstitial pneumonitis and ARDS (Brufsky, 2020; Means, 2020). Increased cell membrane ACE2 receptor levels are found in patients with diabetes (Brufsky, 2020; Means, 2020), which could thus facilitate coronavirus entry into cells and make people with diabetes more susceptible to SARS-CoV-2 infection. In addition, aberrant glycosylation of ACE2 receptor, which can be induced by hyperglycemia, promotes the binding of the SARS-CoV-2 virus to ACE2 receptor and thus increases the severity of COVID-19 disease (Brufsky, 2020). These findings partially explain why hyperglycemic or diabetic individuals have a higher tendency to SARS-CoV-2 infection and a higher disease severity.

ACE2 is also expressed in many other tissues with endothelial cells including the heart and kidneys and, importantly, in insulin producing β-cells (Hussain et al., 2020). SARS-CoV-2 might direct damage β-cells via cell surface ACE2 protein and lead to cell injury and apoptosis causing relative insulin deficiency and acute hyperglycemic state, thereby increasing the risk for developing severe COVID-19 or requiring ICU admission (Hussain et al., 2020). In addition, recent clinical data have highlighted that the activation of coagulation (such as D-dimer) and thrombocytopenia, causing frequent occurrence of coagulopathy in severe COVID-19 patients, is a major extrapulmonary risk for mortality of COVID-19 (McFadyen et al., 2020). Thrombotic manifestations and coagulopathy in severe COVID-19 are both related to SARS-CoV-2 invading endothelial cells via ACE2 receptor (McFadyen et al., 2020). Giving increased ACE2 receptor in diabetes, coagulopathy is much more frequent in COVID-19 patients with pre-existing diabetes. Furthermore, it has been shown that insulin, a medicine for treating hyperglycemia or diabetes, can attenuate ACE2 expression (Muniyappa and Gubbi, 2020), suggesting that well-controlled glycemia may reduce the risk of COVID-19 infection via decreasing the cellular levels of ACE2. In addition, renin‒angiotensin‒aldosterone system (RAAS) inhibitors, which are often used to treat diabetic patients, have been testified to improve outcomes in COVID-19 patients (Koszegi et al., 2019; Pirola and Sookoian, 2020). Thus, increase in ACE2 receptor, through activation of RAAS or others, may be one of the most key mechanism for progression and unfavorable outcome in COVID-19 patients with pre-existing diabetes.

### Increased circulating glucose and glycated hemoglobin

Glycated hemoglobin level is higher in diabetic individuals due to increased circulating glucose level, which could be an important factor for COVID-19 infection and mortality (Liu and Li, 2020; Means, 2020). The surface proteins of SARS-CoV-2 virus could attack heme on the 1-β chain of hemoglobin in red blood cells of individuals with diabetes, dissociating iron to form porphyrin, thereby causing less and less hemoglobin that can carry oxygen, and carbon dioxide ultimately leading to symptoms of respiratory distress (Liu and Li, 2020; Means, 2020). Liu and Li (2020) suggested that deoxyhemoglobin is more vulnerable than oxidized hemoglobin to the surface proteins of SARS-CoV-2 virus, and glycosylated hemoglobin (deoxygenated form) levels are higher in diabetes patients. These findings suggest that patients with hyperglycemia or diabetes may be more vulnerable to SARS-CoV-2 attack due to enhanced glycation of hemoglobin, thus increasing the risk for COVID-19-associated mortality rate. However, it only can be definite that an abnormal accumulation of porphyrins exists in serum from severe COVID-19 patients (San Juan et al., 2020). The pathogenic mechanism of how SARS-CoV-2 virus binds to the 1-β chain of porphyrins of the erythrocytes, leading to release of iron and disturbance of heme metabolism, remains elusive and needs further investigation.

### Diabetes changes lung function and structure

SARS-CoV-2 mainly invades the respiratory tract and lungs, and pulmonary cells represent one of the major cellular sites for coronavirus entry (Hussain et al., 2020). Previously, diabetes is well known to induce pulmonary dysfunction, such as reducing lung volumes and compliance and increasing in airway resistance, which is related to insulin resistance and non-enzymatic glycosylation of lung proteins (Lecube et al., 2017). This may be another risk factor for more severe illness in COVID-19. Thus, the impact of diabetes and obesity on pulmonary dysfunction may explain why people with diabetes are more susceptible to SARS-CoV-2 attack. Furthermore, hyperglycemia induces lung tissue structural changes including collapse of portions of the lung and augmented permeability of the blood vessels (Means, 2020). Therefore, it is conceivable that the lung of diabetic individuals, as a potential target for the harmful effects of hyperglycemia, may accelerate SARS-CoV-2 infection, replication of the virus, and further deterioration.

### Hyperglycemia increases COVID-19 pathogenesis by weakening immune defense system

Clinical and laboratory investigations show that lymphocytopenia, an abnormally low level of lymphocytes in the blood and a marker of severe prognosis, is significantly associated with the severity of COVID-19. Absolute numbers of T lymphocytes, CD4^+^ T cells, and CD8^+^ T cells are all greatly decreased in nearly all COVID-19 severe patients compared with non-severe cases (Guan et al., 2020; Li et al., 2020b). SARS-CoV-2 infection could cause hyper-inflammation in cells, resulting in an excessive activation of macrophages, which can suppress the recruitment of T cells (Iacobellis, 2020). Emerging evidence shows that during the acute phase of infection, SARS-CoV-2 invades CD4^+^ T and CD8^+^ T lymphocytes, leading to cell apoptosis and lymphocytopenia (Angelidi et al., 2020). It has been shown that elevated glucose levels promote SARS-CoV-2 replication in monocytes, resulting in inhibition of T-cell response (Codo et al., 2020). Furthermore, MERS-CoV, which is 50% similar to the genetic sequence of SARS-CoV-2, has been suggested to invade T cells by binding to dipeptidyl peptidase 4 (DPP4) as its cellular receptor and activate the nuclear factor kappa B pathway, leading to the immunity disorders (Iacobellis, 2020). Moreover, diabetes is well known to be associated with dysfunctional innate and adaptive immunity (Donath et al., 2019) and it is thus speculated that impaired immune function in diabetes patients may accentuate SARS-CoV-2 infection and its harmful function.

### Hyperglycemia contributes to abnormal inflammatory responses and immune over-activation

Cytokine storm, the uncontrolled release of high levels of cytokines, is an excessive immune response to external stimuli. Hyperglycemia may also increase inflammatory macrophage (M1) population. Dysregulation of T cells and macrophages leads to increased secretion of inflammatory cytokines and chemokines, triggering cytokine storm (Muniyappa and Gubbi, 2020; Ye et al., 2020). Patients with moderate and severe COVID-19 usually show a marked increase in their (serum) levels of IL-6, TNF-α, IL-2R, IL-10, and other inflammation-related markers such as high-sensitivity C-reactive protein, D-dimer, and ferritin (Ye et al., 2020). These inflammatory-related cytokines may continuously activate the T-helper type 1 cell response, leading to immune over-activation (Huang et al., 2020). In addition, the serum levels of IL-6 and IL-2R in COVID-19 patients are positively correlated with the severity of the disease (Ye et al., 2020), suggesting that the cytokine storm is positively correlated with the severity of COVID-19 infection. This type of abnormal immune response, or cytokine storm, may cause ARDS and multiple organ failure, leading to disease aggravation or even fatality (Ye et al., 2020). Therefore, it may be an important strategy to prevent COVID-19 patients from deterioration and halve the mortality by suppressing the cytokine storm effectively.

**Figure 1:**
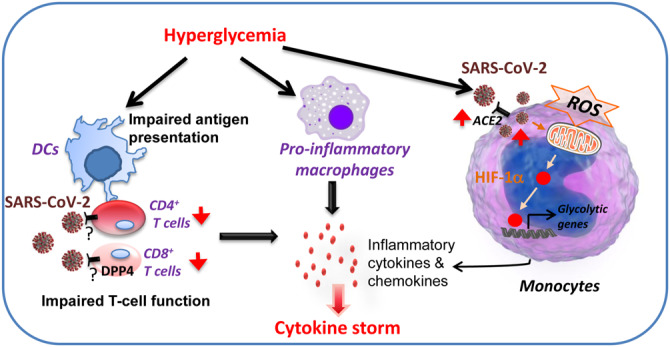
Putative mechanisms by which hyperglycemia induces cytokine storm in COVID-19 patients. T-cell function could be impaired by SARS-CoV-2 infection, which binds to DPP4 on the membrane of T cells, leading to immune suppression with decreased CD4^+^ and CD8^+^ T cells. Poor-controlled blood glucose levels in diabetes may impair antigen presentation in DCs, resulting in T-cell dysfunction. Hyperglycemia may also increase M1 population. Dysregulation of T cells and macrophages leads to increased secretion of inflammatory cytokines and chemokines, triggering cytokine storm. Elevated glucose levels could also promote SARS-CoV-2 replication and ACE2 expression in monocytes, which stabilizes HIF-1α and promotes glycolysis, leading to increased cytokine expression and thus cytokine storm.

Recent researches reveal that cytokine storm emerges in COVID-19 patients with hyperglycemia or T2DM, leading to dysregulation of immune response and ARDS (Ye et al., 2020). Increased levels of chronic inflammatory factors, such as IL-1β, IL-6, TNF-α, and activation of the immune response are frequently found in diabetic patients with poor-controlled blood glucose levels (Apicella et al., 2020). Thus, high blood glucose may promote cytokine storm emerge and immune over-activation, ultimately causing ARDS and multiple organ failure in COVID-19 patients. A latest report shows that elevated blood glucose levels facilitate SARS-CoV-2 replication and ACE2 expression in monocytes accumulated in the lung of COVID-19 patients, which induces mitochondrial reactive oxygen species (ROS) production by stabilizing hypoxia-inducible factor-1α (HIF-1α) and promoting glycolysis (Codo et al., 2020). The increased mitochondrial ROS production promotes cytokine over-release and causes cytokine storm, ultimately leading to inhibition of T-cell response and reduction of epithelial cell survival (Codo et al., 2020). Thus, following SARS-CoV-2 infection, poor-controlled blood glucose in diabetes patients may promote macrophage inflammation and antigen presentation impairment in dendritic cells (DCs), resulting in a great increase in the secretion of inflammatory cytokines and chemokines from immune cells and ultimately cytokine storm and increased mortality (Figure 1). Therefore, targeting HIF-1α may reduce cytokine storm and provide a great therapeutic treatment of COVID-19. Furthermore, effectual blood glucose control to inhibit the cytokine storm and immune dysregulation may provide a reference for the clinical diagnosis and treatment of COVID-19.

### Hyperglycemia induces elevated lactate levels in modulating the inflammatory immune response

**Figure 2:**
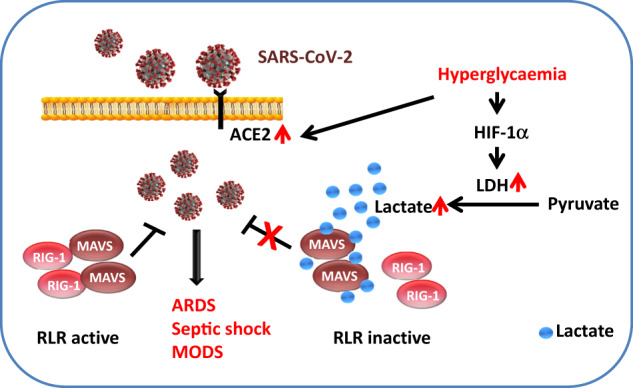
Potential mechanisms by which hyperglycemia facilitates SARS-CoV-2 infection. Hyperglycemia may increase the expression of ACE2, which mediates SARS-CoV-2 infection. Hyperglycemia may also increase lactate production via HIF-1α, which suppresses the innate immune RLR signaling by targeting MAVS, leading to delayed clearance of SARS-CoV-2 and thus severe outcomes in diabetes patients with COVID-19, including ARDS, septic shock, and MODS.

Lactate is previously considered as a metabolic by-product, while cumulative evidence suggests that lactate regulates diverse biological processes such as modulation of the inflammatory immune response (Pucino et al., 2017), suppression of innate immunity (Zhang et al., 2019), and differentiation of T helper cells (Peng et al., 2016). Lactate dehydrogenase (LDH) is a key enzyme that regulates the production and release of lactate, which is markedly increased in adipocytes of obese mice (Petersen et al., 2017). LDH activity is significantly upregulated under hyperglycemic conditions through the accumulation of HIF-1α (Cheng et al., 2019). Moreover, inactivation of LDH can increase production of Type I interferons (IFNs) in response to viral infection (Zhang et al., 2019). Recent clinical and pre-clinical studies reveal that the level of LDH in blood is frequently higher in severe COVID-19 cases (Li et al., 2020b). Thus, it implies that lactate may play a key role in modulating the inflammatory immune response in severe patients with COVID-19 and pre-existing diabetes. However, the potential mechanism underlying lactate action in COVID-19 was little studied. Diabetic individuals are originally more susceptible to bacterial and viral infection (Angelidi et al., 2020). A recent study shows that glycolysis-derived lactate inhibits the retinoic acid-inducible gene I (RIG-I)-like receptor (RLR) signaling by binding to mitochondrial antiviral-signaling protein (MAVS) transmembrane domain directly, ultimately inhibiting IFN production and viral clearance (Zhang et al., 2019; Zhu et al., 2020). Thus, it is speculated that increased lactate production in diabetes may delay the clearance of SARS-CoV-2 by inhibiting the innate immune RLR signaling, leading to a severe or even fatal outcome in diabetes patients with COVID-19 (Figure 2). Further investigations would be important to elucidate the contribution of the pre-existing diabetes and immune dysfunction to COVID-19 severity.

## Concluding remarks

The outbreak of the COVID-19 pandemic has caused a global health crisis of our time. A growing body of clinical evidence shows that patients with pre-existing diabetes are highly susceptible to SARS-CoV-2 infection and its associated mortality. The exact mechanisms linking diabetes and COVID-19 remain to be further elucidated, but available clinical/laboratory observations suggest that hyperglycemia-induced immune dysfunction, cytokines storm, and elevated lactate levels may play critical roles in the severity of COVID-19 in patients with pre-existing diabetes. A large body of evidence shows that hyperglycemia or diabetes may impair immune response mediated by macrophages, monocytes, and DCs, weaken T-cell function, and promote cytokine storm, ultimately resulting in increased susceptibility of SARS-CoV-2 infection and COVID-19-associated mortality. Thus, medications for lowering blood glucose levels, blunting immune suppression and cytokines storm, or suppressing lactate levels would be beneficial for effective therapeutic treatments for COVID-19 in patients with obesity and diabetes.

In addition to common anti-diabetic drugs, some other medicines, such as the traditional Chinese medicine Lianhuaqingwen (LH) and Artemisinine (Qinghao), can or may improve the prognosis of COVID-19 patients with pre-existing diabetes. LH has been exerted broad-spectrum antiviral effects on a series of influenza viruses and immune regulatory effects (Li et al., 2020a). It is also proved that LH can significantly inhibit SARS-CoV-2 replication and may regulate host immune response (Li et al., 2020a). Artemisinine, which is best known for the naturally occurring antimalarial effect, has found its multiple pharmacological functions against inflammation and viral infections (Cheong et al., 2020). It may impact multiple pathways of innate immunity (An et al., 2017). According to clinical treatment results (Lin et al., 2020) and laboratory data (Gendrot et al., 2020), Artemisinine compounds can shorten the treatment time of COVID-19 and improve prognosis. Thus, we speculate that Lianhuaqingwen (LH) and Artemisinine (Qinghao) could be used for the treatment of patients with COVID-19 and pre-existing T2DM. In addition, humanized mouse models of SARS-CoV-2 infection and more other clinical data may provide insights into this disease. Moreover, identification of clinical and/or biochemical parameters and making individual therapeutic recommendations may be helpful for COVID-19 patients, especially for those pre-existing diabetes.

*[This work was partially supported by grants from the National Nature Science Foundation of China (81800758 and 81730022) and the National Key R&D Program of China (2018YFC2000100 and 2019YFA0801903). J.W. drafted the manuscript. W.M. developed the initial concept and framework for the manuscript and revised the manuscript. Both authors contributed to the content, drafting, and critical review of the manuscript.]* 
